# Bioattribution Needs a Coherent International Approach to Improve Global Biosecurity

**DOI:** 10.3389/fbioe.2015.00080

**Published:** 2015-06-01

**Authors:** Randall Steven Murch

**Affiliations:** ^1^School of Public and International Affairs, Virginia Tech Research Center, Virginia Polytechnic Institute and State University, Arlington, VA, USA

**Keywords:** attribution, global biosecurity, investigation, microbial forensics, law enforcement, public health, decision making

## Abstract

The forensic investigation of hoax, suspected or actual biological weapons attacks, and bioproliferation activities is recognized by biosecurity-advanced nations as an important pillar in a national biosecurity program. Some nations have taken this seriously; most others have not or are not aware of the potential. When law enforcement and forensic science investigations are performed in a coordinated manner, decisions assigning attribution are informed and accountability is supported through legal and policy decisions and actions. Incorporating public health investigative and tailored scientific assets makes the system even more effective, dynamic, and robust. Perpetrators and enablers must be held at risk of being brought to justice, or through a policy decision resulting in direct action being taken or sanctions imposed. This paper provides a foundation and path forward to establish substantially expanded capability founded on establishing and leveraging national and regional programs and international agreement that attribution is an important component of biosecurity. Specific forward-looking initiatives will be recommended and discussed.

## Introduction

An effective and agile investigative capability supported by science, other complementary technical support, and information sharing, is an essential aspect of any national biosecurity program as well as global biosecurity (Murch, 2001, 2003, 2011a,b, 2014; Budowle et al., 2003; Bush, 2004; Obama, 2009). Those that perpetrate bioterrorism and bioproliferation must be held at risk for legal prosecution or actions taken as a result of policy decisions. Getting caught and being held accountable, or the credible threat thereof, “raises the bar” for those who engage illicit activities related to biowarfare, bioterrorism, or bioproliferation. The greater the belief of and risk to adversaries that they will be identified, apprehended, and prosecuted either through legal or policy decisions, the more likely this will be factored in the decisions and choices they make.

Greater global cooperation should be established in this arena. Nations could establish tailored programs or cooperate in regional consortia to share benefits and costs, or a sufficient number of countries could to provide adequate coverage for the rest as needed. If this vision is embraced, the first step toward the goal of a more robust global investigation and attribution capability would be specific efforts for building cooperation, engineering consensus, and cooperation on investigative capabilities, legal requirements, and scientific collaborations.

There is no one program that can provide an impervious biodefense or biosecurity. However, a well-crafted “kit of tools,” which effectively enhances anticipation, prevention, preparedness, response, recovery, and attribution can provide a defense system, which “raises the bar” for adversaries and those who assist them. Investigation and attribution are sometimes forgotten as important tools in the kit. This paper focuses on the latter, specifically investigation toward attribution, and argues that substantial expansion of attribution-informing programs would significantly improve biosecurity at a global scale.

## Background

In the United States and other countries, bioterrorism risk mitigation, preparedness, and response are reliant on a number of complementary and layered biosecurity and biodefense programs. In the U.S., this has included developing and stockpiling advanced medical countermeasures, improving emergency response, fostering responsible science, and ensuring safe and secure research in several hundred higher level biosafety level (BSL) laboratories. Singly or taken together, these are not a perfect solution against criminal and terrorist acts. Insider personnel threats, misuse of science, misappropriation of source materials, the threat, intention and acquisition, development, test and use of dangerous pathogens and toxins as weapons by non-state and state actors, and even false threats, must still be taken seriously. Those measures, which increase the likelihood that those who conduct illegal activities will be held at risk of legal or policy action, are also necessary.

At the core of such a “whole of government” investigative response also is the collaborative relationship between public health and law enforcement for executing concomitant public health and criminal investigations (Goodman et al., 2003). Such a program has existed in the U.S. since 1970s, but since 1996 was greatly strengthened and expanded and has provided value almost many cases and events. Since then, the program has undergone considerable expansion with respect to advancing investigative and scientific capabilities, expanding the organizations involved and involvement of policy in addition to criminal justice and law. Since that time, some other countries have created their own programs (Commonwealth of Australia, 2009, 2010; Government Offices of Sweden, 2011; Public Health Agency of Canada, 2015) including sometimes benefiting from engagement with the U.S. From these early efforts in the U.S., the field of microbial forensics was born, which has since become a recognized subdiscipline of microbiology at the intersection with forensic science.

In the years prior to the U.S. program’s creation, laws were enacted to enhance the prosecution of the unauthorized possession, development, or use of biological weapons and toxins, which enabled investigation and prosecution (United States Code, 1989). The investigations of the anthrax attacks of 2001 (Murch, 2011b) were complex, frustrating to many, sometimes seemingly politically driven, and not fully enabled with all the tools required for rapid resolution. However, the fact that reasonably mature investigative capabilities, some existing analytic methods and emerging scientific resources at the time (National Research Council, 2011), and established legal instruments were in place enabled the investigations toward the prosecution (United States Department of Justice, 2010). The technical investigative capabilities have since advanced further (Federal Bureau of Investigation, 2015).

A global BW investigative and forensic program may not precisely mirror the program of any one country. However, much could be learned and leveraged from existing programs to establish a robust system of aptly sized and tailored national and regional capabilities, which provide global coverage.

## Context

There are a several key components of a bioterrorism–bioproliferation forensics and attribution program that the reader should become familiar with.

### Law enforcement investigation

Law enforcement investigation pertains to the application of resources under legal authority, which results in the determination that an event of interest (i.e., criminal) has occurred, follows a logical and defensible path to identifying those responsible (“attribution”), and their methods, means, and motives, and provides a prosecutable case based on admissible evidence, ideally through equitable legal process and decision making. These investigations support actions through criminal justice systems (e.g., prosecutions).

### Attribution

Attribution in the legal and policy contexts refer to “who did it.” Attribution is informed by investigative, scientific, and other information and is a qualitative judgment by the appropriate authority. The “attribution decision” precedes further decision making with respect to what, if any, actions to take as a result of the type and impact of the events at issue. Legal attribution decisions can use existing decision frameworks, but with policy decisions, at least in the U.S., such does not exist. Exoneration is considered to be equally important. Scientific attribution is taken to mean “assignment of a *sample of questioned origin* to *a source of known origin* to a *high degree of scientific certainty* (at the same time excluding origination from other sources). The scientific analytical and interpretive activities, which seek scientific attribution, inform legal and policy questions that can lead to identifying perpetrators and enablers. Depending on the type and quality of the forensic evidence and what it is capable of providing, the science can be very informative, definitive, and buttress either the case of prosecution (seeking conviction and punishment) or defense (exoneration, acquittal).

### Public health investigation

Public health investigation is the process that health authorities undertake in response to a disease outbreak to identify the causative agent, source, and spread of the outbreak to minimize its effects. From the biosecurity perspective, resolving with high confidence whether or not the outbreak manifested as a result of a natural, accidental, or deliberate event is crucial. This is also the nexus of the relationship between public health and law enforcement; and neither can resolve the origination of the outbreak without the other. Public health seeks to protect the health and safety of the public. Law enforcement seeks to protect the public by preventing, interdicting, mitigating, responding to, and bring resolution to criminal (and terrorist) acts. The approaches and science used by both domains are similar, though for different purposes.

### Forensic science

Forensic science can be defined as “the analysis and interpretation of physical evidence to determine its relevance to criminal motives, intentions, plans, events, persons of interest and perpetrators, places of interest including scenes of crime, and means to include tools, methods, and processes.” Forensic science includes the proper collection, preservation, transport, storage, analysis, interpretation and reporting of evidence, and the required administration, security, and quality management. Classical forensic disciplines include pattern analysis (e.g., fingerprint), biology (e.g., human DNA analysis), trace evidence (e.g., hairs and fibers), toxicology (i.e., drugs and poisons), digital evidence (e.g., computer media), and pathology (i.e., manner and cause of death). Validated techniques, methods, and protocols, which meet or exceed both scientific and legal standards, are required. In the U.S. and elsewhere, some forensic science is being called into question because the science and claims about it have been found to be weak, unsupported, overstated, and applied by those without proper credentials (National Research Council, 2009). Hence, in at least the U.S., there is pressing interest for improvements to many areas of forensic science and practice and its use in legal processes.

### Microbial forensics

Microbial forensics is defined as “the development and application of forensic science and necessary sciences to investigative problems involving biological threat agents, their by-products (toxins), and associated physical evidence.” In essence, microbial forensics seeks to help answer: “What was the agent used and its source? How was it prepared and disseminated? Where and how did the illicit activity take place? Who was involved?” Microbial forensics leverages a host of other basic and applied sciences that are not common to traditional forensic sciences; e.g., microbiology, microbial ecology, public health epidemiology, microbial genomics, and bioinformatics, toxicology, and process engineering. Microbial forensics incorporates the investigative, forensic, and legal perspectives and requirements to answer relevant investigative and legal questions. Leaders in microbial forensics are very mindful of these issues and activities to improve forensic science broadly. It requires specialized expertise, training, equipment, and instrumentation, and facilities, which are more akin to public health and infectious disease laboratories, but also with the capacity to exploit physical evidence for classical purposes that is or could be contaminated with hazardous materials. Since its origins, microbial forensics has evolved considerably based on new science, and the experience garnered from prior events such as the anthrax mailing attacks in the U.S. in 2001 (Breeze et al., 2005; Budowle et al., 2010; Cliff et al., 2012; National Research Council, 2014). The best quality of science that meets or exceeds scientific and legal standards or policy expectations is sought (Budowle et al., 2008; Murch and Bahr, 2010), even with advanced technologies (Budowle et al., 2014).

## Preparing and Integrating Essential Elements for Effective Programs

The design, development, validation, establishment, and exercising of capabilities should occur before they are needed. During a crisis, poor planning and preparation will likely result in poor performance, complications resulting in delays, bad outcomes, or even overall failure. Much of this can be avoided by initially conducting a thorough and objective assessment of what capabilities exist and what is needed, and then laying out and acting on a plan to get the current state to the desired state in a rational, cost-effective manner. Awareness training for and exercising by key legal and policy offices should be provided in advance to minimize impedances on operations and decision making.

Key components are:

*Properly Staffed Teams, Coordinated Investigation, Information Access, Analysis, and Sharing*: Law enforcement and public health investigative teams require individuals that possess strong professional credentials and experience in their respective professions and disciplines, and experience in complex investigations, including joint investigations. These teams should have established and tested investigative protocols; coordination and cross communication between the respective teams, respective lines of command, control and communication, and protocols for engagement with key stakeholders; and, all necessary on-demand information and knowledge resources.
*Technical Forensic Capabilities*: Forensic hazardous materials (hazmat) sampling and contaminated traditional evidence collection must occur in an effective, safe, and secure manner. Evidence must be packaged, documented, and transported in an appropriate manner to maintain the chain of custody and evidence integrity. Necessary equipment and logistics must be available and used to meet regulations for hazmat transport. Fully equipped and configured laboratories staffed by the appropriately educated and certified personnel should be available to conduct required analyses using validated methods under a comprehensive quality program. These experts render the accurate and defensible reports and opinions that answer or inform priority investigative, forensic, legal, and policy questions.
*Legal Process and Decision Making:* Laws must be in place to allow the prosecution and punishment of illegitimate acquisition, development, use, and proliferation of dangerous pathogens and toxins. Further, the criminal jurisprudence system of a country must be able to recognize, react to, and resolve violations of law. Given that cases involving dangerous pathogens and toxins have a number of complexities and uncertainties to contend with, lawyers and judges must be aware of these and understand how the law and legal procedure applies. In some countries, this extends to complex science that must be applied, interpreted, and reported, which then must be conveyed in the language and logic of the courts to inform decisions, including those of guilt or innocence. Forensic experts must be trained to and communicate complex science in an understandable, relevant manner, and ensure that decision makers understand the value, weight, attributes, limitations, and uncertainties of the science and practice.
*Policy Process and Decision Making:* Policy formulation and decision making is different than the legal process, though it may be influenced by law as well as politics. That said, the investigation, and particularly the supportive scientific activities conducted should be thorough, timely, objective, informative, and defensible with the understanding that policy makers will consider and act on what is provided as they see fit. In keeping with good science and professional integrity, nevertheless, policy makers, and politicians should be provided relevant, complete, accurate, reliable, testable, defensible information, and conclusions from the technical experts to use in deliberations and decision making.

## Moving Forward: Building the Base toward Broad Implementation

The following are key steps that, if undertaken, would move nations, regions, and perhaps our global society toward and to the goal of a coherent investigative, technical, legal, and policy enterprise for bioattribution.

### Recommendation 1: Increasing awareness and engagement

Regional security cooperation conferences should be convened, which include engaging the international law, diplomatic, policy, technical and investigative communities on the attribution of biological warfare, bioterrorism, and bioproliferation. The foci of these meetings would be to provide awareness and education, and allow for structured discussions on investigative priorities and processes, interagency coordination, technical aspects, and decision making. Participants would consider: (a) the importance of and current and desired capabilities to properly investigate suspicious disease outbreaks; (b) whether having an improved capacity to investigate and inform decision making should be a priority; and (c) if the answers to the first two sets indicate positive interest, then how best to organize, resource, evolve, and sustain the desired capability either within country, as a regional construct or through outsourcing it. Tabletop exercises would be an excellent method to immerse participants in applying the knowledge that they have gained to quickly gain a deeper understanding. Discussions on national and international legal and policy requirements to declare attribution and to support actions could also take place. Joint statements of need or draft agreements and “paths forward” could also result from such meetings.

### Recommendation 2: Country assessments to inform investments

Using external or indigenous experts “country assessments” could be performed using a tailored “systems analysis” approach. The requestor would articulate what they seek to establish in a prescribed format. A team of vetted experts in the appropriate disciplines would help to objectively determine what exists, what is needed, how to, and what is required to achieve the desired state and the timeline. The results and recommendations would be provided to the requestor for consideration and action. The experts assisting could be available as reachback support.

### Recommendation 3: Integrated training and cross-community exercises

Various types and levels of training could be provided to requesting nations based on need, existing capacity, available resources, and desired end-state. Based on prior analysis, the training for each requesting country would also seek to facilitate the integration of law enforcement and public health investigation and their respective scientific and technical elements, and criminal justice and policy stakeholders. Tabletop or field exercises would be executed to assist in the requesting nation with burnishing its newly acquired knowledge or capability. The “train-the-trainer” perspective would be embedded throughout, with reachback support available from the original training staff as needed.

### Recommendation 4: Pilot (demonstration) efforts

At least four areas exist for which focused, limited-term projects could be designed and executed to catalyze near- and longer-term success.

- Deeper, hands on training for new technical capabilities could be provided to nations that have or can quickly implement basic capabilities. More sophisticated sample collection, and advanced instrumentation and analytic methods could be made available.
- Collaborative research by multinational teams focused on “microbial forensics grand challenges” (National Research Council, 2014), which would provide fundamental leaps in knowledge and capability could be funded and pursued to galvanize attention on these topics and contribute to substantial advancements in knowledge and technology that would benefit microbial forensics and closely related fields.
- International agreement on establishing and accepting guidelines and standards for the practice of microbial forensics would be core to a confidence-building quality management system governing laboratory operation and performance, as it is with other fields. One of the most common bodies of standards used in forensic science is International Standards Organization (ISO)/IEC 17025 standards, General Requirements for the Competence of Calibration/Testing Laboratories (International Standards Organization, 2010), against which laboratories are inspected in order to achieve accreditation. Certification of practitioners, accreditation of laboratories, and validation of methods would be three priority topics. The most widely accepted approaches and best practices from forensic science, microbial forensics, public health epidemiology, environmental health and microbiology, food safety, and other pertinent fields could also contribute to guidelines or standards for bioterrorism and bioproliferation investigations. One or more consensus reports with courses of action would result.
- Developing and testing attribution decision frameworks would develop from structured conversations among legal and policy experts, augmented by technical experts, that would be the precursor to developing a global conversation regarding what should be expected regarding the type, quality, quantity, and value of information that is required to make an accurate, reliable, and defensible attribution decision. Thus, decision frameworks could be established and vetted for broad consideration. These fora could also be leveraged for discussions on bioattribution and deterrence, which have received no serious attention.

If the above were undertaken and sustainability was determined to be advantageous, a global strategy and plan could be developed and enterprise leadership could be established. These could occur before or after the aforementioned recommendations were undertaken. Other subsequent opportunities would be obvious and realized as well. Adding robust attribution capabilities will enhance the global biosecurity regime. A more biosecure future awaits us.

## Conflict of Interest Statement

The author declares that the research was conducted in the absence of any commercial or financial relationships that could be construed as a potential conflict of interest.
